# Prevalence and Associated Factors of Intestinal Parasitic Infections and Undernutrition Among Elementary School Children in Zenzelima Town, Northwest Ethiopia: A School‐Based Cross‐Sectional Study

**DOI:** 10.1002/hsr2.70964

**Published:** 2025-07-02

**Authors:** Abera Ademasu Birhanu, Mulat Yimer, Habtu Debash, Megbaru Alemu Abate

**Affiliations:** ^1^ Bahir Dar Health Science College Bahir Dar Ethiopia; ^2^ College of Medicine and Health Sciences Bahir Dar University Bahir Dar Ethiopia; ^3^ College of Medicine and Health Sciences Wollo University Dessie Ethiopia; ^4^ School of Public Health The University of Queensland Brisbane Australia

**Keywords:** Ethiopia, intestinal parasites, school children, undernutrition, Zenzelima

## Abstract

**Background and Aims:**

Intestinal parasites and malnutrition are major public health challenges in Ethiopia, highlighting the need for local epidemiological data to inform effective prevention and intervention strategies. This study aimed to assess the prevalence of intestinal parasitic infection, undernutrition, and their associated risk factors among elementary school‐aged children in Zenzelima town, Northwest Ethiopia.

**Methods:**

A school‐based cross‐sectional study was conducted from January to May 2022. Systematic random sampling was used to select study participants. Sociodemographic and risk factor data were collected using a structured questionnaire. Height and weight measurements were taken using a meter and calibrated balance. Fresh fecal samples were collected and processed using wet mount and formol‐ether concentration techniques.

**Results:**

Of 405 study subjects, 201 (49.6%) were infected with one or more intestinal parasites. The predominant parasite was hookworm (22.2%), followed by *Giardia lamblia* (19.8%) and *Entamoeba histolytica* (13.6%). Habits of eating raw vegetables/fruits (adjusted odds ratio [AOR] = 2.03; 95% confidence interval [CI]: 1.330–3.11), having irregular use of closed shoes (AOR = 1.86; 95% CI: 1.09–3.47), and exhibiting the habit of open‐field defecation (AOR = 3.07; 95% CI: 2.00–4.71) were identified as independent predictors of intestinal parasitic infections. Alternatively, the overall prevalence of undernutrition among children was 58.3%, consisting of 49.1% for wasting, 25.9% for stunting, and 14.6% for underweight. Meal frequency at most three times a day (AOR = 3.24; 95% CI: 1.98–5.32) and infected with intestinal parasites (AOR = 1.84; 95% CI: 1.19–2.83) were strongly associated with undernutrition.

**Conclusion:**

The prevalence of intestinal parasitic infections and undernutrition was high among school children in Zenzelima, Ethiopia. In this study, eating raw vegetables/unwashed fruits, irregular use of closed shoes, and open‐field defecation were statistically significant risk factors for intestinal parasitic infections. Moreover, low meal frequency and intestinal parasitic infections were significantly associated with undernutrition. These results highlight the need to strengthen integrated strategies to reduce both intestinal parasitic infections and undernutrition.

## 1 Introduction

Intestinal parasitic infections are major public health problems worldwide. According to the World Health Organization (WHO), 1.5 billion people of the world population have intestinal parasirtic infections, mainly the soil‐transmitted helminthes such as *Ascaris lumbricoides* (*A. lumbricoides*), *Trichuris trichiura* (*T. trichiura*), and hookworms [1]. Moreover, *Giardia lamblia* (*G. lamblia*), *Entamoeba histolytica* (*E. histolytica*), and *Cryptosporidium* are the most common intestinal protozoal infections in developing countries [2]. These infections significantly contribute to morbidity, particularly in children, leading to malnutrition and developmental issues [3]. Additionally, intestinal parasitic infections result in substantial economic costs, emphasizing the urgent need for effective public health interventions to control and prevent these infections [4]. In developing nations, factors such as poor sanitation, overcrowding, and climate conducive to parasite survival contribute significantly to transmission [5, 6]. School‐aged children face a higher risk of infection due to frequent soil contact and less health awareness [6, 7]. Infected children often suffer from physical, nutritional, and cognitive impairments due to nutrient malabsorption and reduced dietary intake, which can lead to stunted growth, general weakness, and poor academic performance [5, 8].

Undernutrition also poses a severe problem in Africa and Asia, where stunting and thinness affect 20%–30% and 35% of school children in Africa, respectively [9]. While Millennium Goals targeted reducing undernutrition, the issue remains high in sub‐Saharan Africa [10]. Therefore, undernutrition remains a major challenge for African children. In Ethiopia, pooled stunting and wasting prevalence among school children were 23.1% and 22%, respectively [11]. Potential contributing factors for malnutrition include meal frequency, family size, age, sex, and household income [11]. In developing nations, intestinal parasites and malnutrition are often coexisting [12]. Intestinal parasitic infections and malnutrition among children have a synergistic relationship. Parasitic infections can induce malnutrition by hindering nutrient absorption, while malnutrition compromises immunity, heightening vulnerability to parasites [13, 14]. This self‐reinforcing cycle intensifies the severity of both issues over their individual impacts. Considering parasites and malnutrition jointly represent leading global health challenges, their synergistic interplay is thought to exacerbate poor general well‐being in school‐aged children [12, 15, 16]. Therefore, integrated interventions are required to break this cycle of deteriorating conditions to meaningfully improve children's well‐being.

Although several studies in Ethiopia and other low‐income countries have documented the burden of intestinal parasitic infections and malnutrition among school‐aged children [17, 18, 19, 20], many are limited in scope, focusing primarily on either the prevalence or associated risk factors without adequately examining the interplay between infections and nutritional status. Furthermore, most available studies are conducted in rural settings, with limited attention to semi‐urban or peri‐urban contexts where health determinants may differ [21, 22]. Despite Ethiopia's efforts in health education, deworming, and nutrition programs, intestinal parasites and malnutrition remain significant public health challenges, especially among vulnerable populations like elementary school children [23]. In developing countries like Ethiopia, intestinal parasitic infections and malnutrition are closely linked, often exacerbating one another and leading to severe health consequences. Previous studies have reported high rates of intestinal parasitic infections among children in various regions of Ethiopia [11, 12, 24]. However, there remains a significant knowledge gap regarding how socio‐environmental factors (e.g., water source, hygiene practices, sanitation access, household size) interact to influence the co‐occurrence of these health burdens in specific local contexts. To date, no published studies have assessed the dual burden of intestinal parasitic infections and malnutrition in Zenzelima town, despite its proximity to areas with reported high prevalence of IPIs and undernutrition. While prior research has identified various socioeconomic determinants of health, there is limited understanding of how these factors interact within the Zenzelima community. Moreover, disaggregated data at the town level are essential for designing context‐specific interventions, as national surveys often lack the granularity needed to reflect local variations in disease burden and risk factors [25].

This study aims to fill these significant knowledge gaps by examining the prevalence and associated factors of intestinal parasitic infections in the study area. Despite existing research, comprehensive data on how specific behavioral and environmental factors contribute to infection rates remains limited. Therefore, this study aims to investigate the social and environmental determinants of both intestinal parasitic infections and malnutrition, providing a comprehensive view of the contributing factors. The findings will be critical for local health authorities and policymakers, as identifying the prevalence and associated risk factors will help design targeted health education, deworming programs, and nutritional interventions tailored to the community's needs. Additionally, this study will serve as a baseline for future research and health initiatives, enabling continuous monitoring of trends in intestinal parasitic infections and malnutrition over time. Given the pressing need to understand the prevalence and risk factors of these issues in Zenzelima town, Northwest Ethiopia, this study seeks to address the existing knowledge gap. Ultimately, it aims to contribute to improved health outcomes for children in the region and inform public health strategies that can effectively tackle these interlinked challenges.

## 2 Materials and Methods

### 2.1 Study Area

This study was conducted at Zenzelima Elementary School in Zenzelima town. Zenzelima is a small town located in the West Gojjam Zone of the Amhara Region in Ethiopia. It is situated about 430 km from the capital, Addis Ababa. With a population of around 15,000 people, it has a cool and temperate climate at an altitude of 2347 meters above sea level. Agriculture is the main economic activity, with crops like teff, barley, wheat, potatoes, and peas grown, as well as livestock rearing. Basic health and educational services are available but limited, with a health center providing outpatient care and a few primary schools. There are a few primary schools in the town as well as a junior high school that teaches up to Grade 8. Literacy levels remain low due to limited educational opportunities. Access to safe water, sanitation, and solid waste management is challenging, as many residents continue using open fields and streams. Overall, Zenzelima has a predominantly rural setting where subsistence farming and pastoralism are the main livelihoods, facing difficulties related to infrastructure, health services, and water/sanitation.

### 2.2 Study Design and Period

A school‐based cross‐sectional study was conducted from January to May 2022 to determine the prevalence of intestinal parasites, undernutrition, and associated factors among school children.

### 2.3 Inclusion and Exclusion Criteria

Children of school‐going age in Grades 1 through 8 who were present during the study period were eligible for inclusion, provided their parents or guardians (for younger children) signed a written consent form confirming willingness to participate. However, the inabilities to properly collect a stool sample, incomplete responses on the sample collection form by students or guardians, and the use of antiparasitic drugs within the previous 3 weeks were excluded.

### 2.4 Variables

The dependent variables include the prevalence of intestinal parasitic infections and undernutrition, while independent variables encompass socioeconomic factors, behavioral factors (dietary habits, hygiene practices), environmental factors (living conditions, sanitation access), and demographic factors (age, gender) [7, 26].

### 2.5 Sample Size and Sampling Techniques

The sample size was calculated using the single population proportion formula, with the following assumptions: prevalence (p) of 50%, margin of error of 5%, and a 10% nonresponse rate. This yielded a required sample of 422 children. There were a total of 2121 students enrolled in Grades 1 through 8 during the study period, distributed across 36 classes with an average of 59 students/class. Students were stratified by grade level proportionate to enrollment. Final selection of participants was conducted through systematic random sampling using the class rosters as the sampling frame.

### 2.6 Data Collection and Laboratory Procedures

#### 2.6.1 Sociodemographic Data and Risk Factors

A structured questionnaire was developed based on known risk factors, translated into Amharic, and back‐translated into English. Student ages were obtained from the school registration book and confirmed with parents or guardians. Data collectors inquired about the use of protective shoes and hand washing before meals. They also assessed finger cleanliness and overall personal hygiene for each student. Additional data collected included family size, literacy rates of parents, meal frequency, and information on other independent variables. This questionnaire was used to interview participants or the parents of younger children, gathering sociodemographic data and information on behavioral and hygiene practices. Data collection was conducted by trained individuals with relevant experience under the continuous supervision of the investigator.

#### 2.6.2 Stool Sample Collection and Processing

Approximately 2 g of stool were collected from each participant using a labeled, wide‐mouthed, leak‐proof container. Samples were transported immediately to the Zenzelima health center laboratory for processing. Approximately 2 mg of fresh stool was emulsified in a saline drop on a slide, covered, and examined microscopically at 10× and 40× objectives. Additionally, about 0.5 g of stool was mixed with 10 mL of saline, strained, and centrifuged. The supernatant was removed after adding 2.5 mL of 10% formaldehyde and 1 mL of ether. A drop of the sediment was then covered and examined microscopically following the formol‐ether concentration technique [27].

#### 2.6.3 Anthropometric Measurements

Anthropometric measurements were taken by a trained nurse using standard procedures. Height was measured to the nearest 0.1 cm using a stadiometer with shoes removed and feet flat on the ground. Weight was also measured to the nearest 0.1 kg using a digital scale with light clothing and no shoes. The WHO AnthroPlus software was used to classify nutritional status. This involved calculating *z*‐scores for height‐for‐age (HAZ) and body mass index‐for‐age (BAZ) based on the WHO standards. Children with *z*‐scores below −2 SD were classified as underweight (WHZ < −2 SD), stunted (HAZ < −2 SD), and wasted (BAZ < −2 SD). Undernutrition is defined as a condition where a child is classified as underweight, stunted, or wasted. This term encompasses various forms of nutritional deficiencies impacting growth and overall health [28].

### 2.7 Quality Assurance

Several quality control measures were implemented. All data collectors received training, and data collection was supervised daily by the principal investigator. The questionnaire was translated into the local Amharic language and back‐translated into English to ensure consistency. It also underwent pretesting. Anthropometric equipment was calibrated every five measurements to control inaccuracies. Laboratory procedures strictly followed standard operating procedures. This was to ensure high‐quality data by controlling for potential errors and biases during data collection and analysis processes.

### 2.8 Data Processing and Analysis

The data were checked for completeness and entered into the Statistical Package for the Social Sciences (SPSS) version 23 for analysis. Descriptive statistics, including frequencies, prevalence, and means, were used to summarize the data. The WHO AnthroPlus software version 1.0.4 was utilized to convert anthropometric measurements into nutritional indices. Children's nutritional status was assessed based on weight‐for‐age, HAZ, and weight‐for‐height. Risk factors for the acquisition of intestinal parasitic infections and undernutrition were analyzed using bivariable and multivariable logistic regression to evaluate the associations between various independent variables and the dependent variables. A priori significance was set at *p* < 0.05, and two‐sided tests were employed for this analysis. Variables with a *p* < 0.25 in the bivariable analysis were included in the multivariable logistic regression. Adjusted odds ratios (AOR) were calculated with a 95% CI to assess the strength of these associations while controlling for potential confounders, with a *p* < 0.05 considered statistically significant.

#### 2.8.1 Ethical Approval and Consent to Participate

Ethical approval was secured from the Research and Ethical Review Committee of Bahir Dar University, College of Medicine and Health Sciences. Permission was also obtained from the Amhara Public Health Institute. The aim of the study was explained to the study participants and their parents or guardians. Students were involved in the study after receiving written informed consent from their families/guardians and the school principal. Confidentiality of the data was maintained. Children who tested positive for intestinal parasitic infections or were malnourished were referred to the Zenzelima Health Centre for treatment, counseling, and appropriate management. The study adhered to all principles of ethical research conduct as outlined in the Declaration of Helsinki.

## 3 Results

### 3.1 Sociodemographic Characteristics

A total of 405 elementary school children aged 6–19 years participated in the study. Among these participants, 208 (51.4%) were female, and 207 (51.1%) were aged between 11 and 15 years. The majority of the children's mothers, accounting for 72.1%, had no formal education. Furthermore, 253 (62.5%) of the study participants resided in rural areas (Table 1).

**Table 1:** Sociodemographic characteristics of elementary school children in Zenzelima town, Northwest Ethiopia, 2024.

Variables	Categories	Frequency (*n*)	Percentage (%)
Age groups (years)	6–10	191	47.2
	11–15	207	51.1
	16–19	7	1.7
Sex	Male	208	51.4
	Female	197	48.6
Level of grade	1–4	211	52.1
	5–8	194	47.9
Residence	Urban	152	37.5
	Rural	253	62.5
Family size	≤ 5	201	49.6
	> 5	204	50.4
Literacy rate of mother	Secondary school and above	4	1.0
	Primary school	109	26.9
	Illiterate	292	72.1
Mother's occupation	Government employee	4	1.0
	Private employee	5	1.2
	Merchant	51	12.6
	House wife	313	77.3
	Casual labourer	32	7.9
Literacy rate of the father	Secondary school and above	10	2.5
	Primary school	228	56.3
	Illiterate	167	41.2
Father occupation	Government employee	6	1.5
	Private employee	7	1.7
	Merchant	63	15.6
	Causal laborer	46	11.4
	Farmer	283	69.9

### 3.2 Prevalence of Intestinal Parasitic Infections and Associated Factors

The overall prevalence of intestinal parasites in this study was 49.6% (95% CI: 44.4–54.8), indicating that children were infected with at least one species of intestinal parasite. The rates of single, double, and triple infections were 139 (34.3%), 56 (13.6%), and 6 (2.0%), respectively. In total, eight different types of intestinal parasites were identified. The three most common parasites detected were hookworm (22.2%), *G. lamblia* (19.8%), and *E. histolytica* (13.6%) (Table 2).

**Table 2:** Prevalence of intestinal parasitic infections among elementary school children in Zenzelima town, Northwest Ethiopia, 2024.

Variable	Categories	Frequency (*n*)	Percent (%)
Types of intestinal parasites	Hookworm	90	22.2
	*Ascaris lumbricoides*	11	2.7
	*Strongyloides stercoralis*	1	0.2
	*Schistosoma mansoni*	25	6.2
	*Hymenolepis nana*	6	1.5
	*Enterobius vermicularis*	2	0.5
	*Giardia lamblia*	80	19.8
	*Entamoeba histolytica/dispar*	55	13.6
Types of infections	Single infection	139	34.3
	Double infection	56	13.6
	Triple infection	6	2.0
Overall		201	49.6

In this study, students who consumed raw or unwashed vegetables and fruits (AOR = 2.03; 95% CI: 1.33–3.11), had irregular use of closed shoes (AOR = 1.86; 95% CI: 1.09–3.47), and exhibited the habit of open‐field defecation (AOR = 3.07; 95% CI: 2.00–4.71) were identified as independent predictors of intestinal parasitic infections (Table 3).

**Table 3:** Binary logistic regression analysis of determinant factors associated with intestinal parasitic infections among elementary school children in Zenzelima town, Northwest Ethiopia, 2024.

Variables	Categories	IPs		Crude OR (95% CI) *p*	Adjusted OR (95% CI) *p*
		Pos	Neg		
Age (years)	6–10	94	97	0.73 (0.158–3.34) 0.68	
	11–15	103	104	0.98 (0.66–1.45) 0.91	
	16–19	4	3	1	
Sex	Male	95	113	0.72 (0.49–1.07) 0.10	
	Female	106	91	1	
Grade level	1–4	103	108	0.93 (0.63–1.38) 0.73	
	5–8	98	96	1	
Residence	Urban	81	71	1	
	Rural	120	133	0.79 (0.53–1.18) 0.25	
Family size	≤ 5	96	105	1	
	> 5	105	99	1.16 (0.79–1.71) 0.46	
Literacy rate of mother	≥ Secondary school	3	1	1	
	Primary school	48	61	1.34 (0.86–2.09) 0.19	
	Illiterate	150	142	0.35 (0.04–3.43) 0.37	
Hand washing before and after eating	Yes	144	178	1	1
	No	57	26	2.71 (1.62–4.527) 0.02	2.61 (0.99–4.51) 0.06
Eating raw fruits and vegetables	Yes	116	80	2.12 (1.42–3.15) 0.001	**2.03 (1.33–3.11) 0.001**
	No	85	124	1	1
Source of drinking water	Pipe	87	98	1	
	River	8	3	0.39 (0.10–1.50) 0.17	
	Well or stream	106	103	1.16 (0.78–1.72) 0.47	
Open field defecation	Yes	141	84	3.36 (2.23–5.06) 0.001	**3.07 (2.00–4.71) 0.001**
	No	60	120	1	1
Hand washing after toilet/defecation	Yes	89	91	1	
	No	112	113	1.01 (0.69–1.50) 0.95	
Presence of toilet	Yes	116	131	1	
	No	85	73	1.32 (0.88–1.96) 0.18	0.66 (0.39–1.12) 0.12
Finger nail trim	Yes	183	184	1	
	No	18	20	0.90 (0.46–1.77) 0.77	
Regular wearing of closed shoes	Yes	21	41	1	1
	No	180	163	2.16 (1.22–3.80) 0.01	**1.86 (1.09–3.47) 0.03**
Playing with soil	Yes	12	16	0.75 (0.34–1.62) 0.46	
	No	189	188	1	
Eating soil	Yes	6	7	0.87 (0.29–2.62) 0.80	
	No	195	197	1	
Swimming habit	Yes	97	102	0.93 (0.63–1.38) 0.73	
	No	104	102	1	
Nutritional status	Undernourished	133	103	1.92 (1.28–2.86) 0.001	0.82 (0.98–2.81) 0.09
	Normal	68	101	1	1

*Note:* Bold values are statistically significant.

Additionally, hookworm was the most prevalent parasite identified in this study. The multivariable logistic regression analysis conducted to identify the factors influencing hookworm infection revealed that wearing shoes was the sole predictor of hookworm infection (Table 4).

**Table 4:** Binary logistic regression analysis of determinant factors associated with hookworm infections among elementary school children in Zenzelima town, Northwest Ethiopia, 2024.

Variables	Categories	Hookworm		Crude OR (95% CI) *p*	Adjusted OR (95% CI) *p*
		Pos	Neg		
Age (years)	6–10	36	155	0.43 (0.16–1.20) 0.26	
	11–15	51	156	0.27 (0.04–1.77) 0.28	
	16–19	3	4	1	
Sex	Male	50	158	0.94 (0.56–1.58) 0.82	
	Female	40	157	1	
Grade level	1–4	43	168	2.191 (0.79–6.05) 0.13	0.95 (0.570–1.58) 0.84
	5–8	47	147	1	
Residence	Urban	37	115	1	
	Rural	53	200	0.73 (0.44–1.24) 0.24	0.77 (0.47–1.26) 0.30
Family size	≤ 5	42	159	1	
	> 5	48	156	1.06 (0.64–1.75) 0.84	
Literacy rate of mother	≥ Secondary school	1	3	1	
	Primary school	20	89	1.23 (0.68–2.23) 0.77	
	Illiterate	69	223	0.70 (0.06–7.70) 0.49	
Hand washing before and after eating	Yes	65	257	1	
	No	25	58	1.91 (1.06–3.44) 0.03	1.723 (0.983–3.02) 0.07
Open field defecation	Yes	60	165	1.79 (0.97–3.29) 0.06	1.65 (0.991–2.74) 0.06
	No	30	150	1	
Hand washing after toilet/defecation	Yes	44	136	1	
	No	46	179	0.60 (0.33–1.09) 0.95	
Presence of toilet	Yes	52	195	1	
	No	38	120	1.08 (0.57–2.08) 0.81	
Regular wearing of closed shoes	Yes	5	57	1	
	No	85	258	3.16 (1.18–8.43) 0.02	**3.09 (1.19–8.09) 0.02**
Playing with soil	Yes	6	22	1.04 (380–2.853) 0.94	
	No	84	293	1	
Eating soil	Yes	1	12	0.26 (0.03–2.19) 0.21	0.25 (0.03–2.04) 0.19
	No	89	303	1	
Nutritional status	Undernourished	61	175	0.66 (0.38–1.15) 0.14	0.70 (0.45–1.19) 0.18
	Normal	29	140	1	

*Note:* Bold values are statistically significant.

### 3.3 Nutritional Status and Associated Factors

The study also examined the nutritional status and associated factors among elementary school children. The results found that the overall prevalence of undernutrition was 58.3% (95% CI: 53.1–63.0). Specifically, the prevalence rates for wasting, stunting, and underweight were 49.1%, 25.9%, and 14.6%, respectively. Among the undernourished students, 31.4% were exhibited two or more concurrent forms of undernutrition, according to the findings presented in Table 5.

**Table 5:** The prevalence of malnutrition among elementary school children in Zenzelima town, Northwest Ethiopia, 2024.

Nutritional status	Categories		Frequency (*n*)	Percentage (%)
	Malnutrition	Wasted	199	49.1
		Stunted	105	25.9
		Underweight	59	14.6
		≥ 2 forms of undernutrition	127	31.4
		Overall	236	58.3
	Normal		169	41.7

*Note:* Bold values are statistically significant.

The binary logistic regression model result showed that study participants who had a meal frequency of at most three times a day (AOR = 3.24, 95% CI: 1.98–5.32), and those being infected with intestinal parasites (AOR = 1.84; 95% CI: 1.19–2.83) were strongly associated with undernutrition (Table 6).

**Table 6:** Binary logistic regression analysis of determinant factors associated with undernutrition among elementary school children in Zenzelima town, Northwest Ethiopia, 2024.

Variables	Categories	Undernutrition		Crude OR (95% CI) *p*	Adjusted OR (95% CI) *p*
		Yes	No		
Age (years)	6–10	86	105	1.09 (0.24–5.01) 0.91	
	11–15	147	60	0.33 (0.22–1.51) 0.54	
	16–19	3	4	1	
Sex	Male	129	79	1.37 (0.92–2.04) 0.12	1.29 (0.83–2.01) 0.26
	Female	107	90	1	1
Grade level	1–4	94	117	0.29 (0.19–1.45) 0.47	1
	5–8	142	52	1	
Residence	Urban	89	63	1	1
	Rural	147	106	0.98 (0.65–1.48) 0.93	
Family size	≤ 5	109	92	1	1
	> 5	127	77	1.39 (0.94–2.07) 0.10	0.96 (0.62–1.50) 0.87
Literacy rate of mother	≥ Secondary school	2	2	1	1
	Primary school	54	55	1.61 (0.22–11.57) 0.64	1.50 (0.17–13.14) 0.72
	Illiterate	180	112	1.64 (1.05–2.55) 0.03	1.59 (0.97–2.58) 0.06
Literacy rate of father	≥ Secondary school	6	4	1	
	Primary school	135	93	0.91 (0.61–1.36) 0.64	
	Illiterate	95	72	0.88 (0.24–3.23) 0.85	
Meal frequency	> 3	42	65	1	1
	≤ 3	194	104	2.89 (1.83–4.55) 0.001	**3.24 (1.98–5.32) 0.001**
Intestinal parasite	Infected	133	68	1.92 (1.28–2.86) 0.001	**1.84 (1.19–2.83) 0.006**
	Noninfected	103	101	1	1

## 4 Discussion

Intestinal parasites are a significant public health issue in developing countries, with school‐aged children being at particularly high risk, as supported by previous research [29]. The present study was conducted in elementary schools in the town of Zenzelima, Ethiopia, which has a warm climate and other factors conducive to intestinal parasitic infections. Past records from the Zenzelima health center laboratory indicated intestinal parasites were the main cause of morbidity among suspected cases in the study area. This study found the overall prevalence of intestinal parasites among primary school children in Zenzelima was 49.6% (95% CI: 44.2–54.6). This result was comparable to rates found in other studies in Ethiopia, such as 52.4% in Bahir‐Dar town [24], 44.3% in Sekota town [30], 46.5% in Arba Minch town [31], 28.8% in Hakim District, Harari [32] and a pooled prevalence of 46.09% among primary school children across Ethiopia [7].

The current study found the prevalence of intestinal parasites to be higher than some past studies conducted in similar populations, such as 42.9% in Merawi town, Ethiopia [33], 28.9% in Dessie town, Ethiopia [34], 21.5% in Harbu town, Ethiopia [35], and 29.9% in Sebaya, Ethiopia [36]. Lower prevalences were also reported in studies from Kenya (17.3%) [37], Nepal (33%) [38], and Western Iran (9.8%) [39]. However, the prevalence in the present study was lower than others, including 61.7% in Mecha district, Ethiopia [40], 62.3% in Dera District, Ethiopia [41], 57.9% in Jawi town, Ethiopia [42], and 64.6% in Mizan‐Aman city [43]. Higher rates were additionally found in Sudan (87.2%) [44], Madagascar (71.0%) [45], Argentina (78.1%) [15], and Colombia (100%) [46]. These differences may be due to variations in environmental and socioeconomic conditions between locations, as well as disparities in the implementation of primary healthcare, deworming interventions, diagnostic methods, and levels of community hygiene awareness and behaviors. The remarkably high prevalence reported in Colombia could potentially be related to the use of concentrated stool sediments examined microscopically and the Kato‐Katz test.

The intestinal parasite analysis identified several organisms, with hookworm (22.2%), *G. lamblia* (19.8%), and *E. histolytica* (13.6%) being the dominant types detected. This high prevalence of hookworm was comparable with previous findings in Dera District (21.7%) [41], Jawi town (13.8%) [42], and Maksegnit, Northwest Ethiopia [47]. In contrast, a study in Dembecha town identified *A. lumbricoides* as the dominant parasite, followed by hookworms, *T. trichiura*, and *Strongyloides stercoralis* (*S. stercoralis*) [48]. Additionally, a systematic review in Ethiopia found *Entamoeba* spp. to be the most common intestinal parasite, followed by *A. lumbricoides*, hookworm, and *G. lamblia* [7]. The elevated levels of hookworm in the current study may be attributed to a lack of awareness regarding the importance of wearing protective shoes and the magnitude of open defecation practices. Not wearing shoes increases vulnerability to hookworm infection, as supported by other research [49]. This highlights the need for targeted health education and interventions to improve personal hygiene practices and reduce the incidence of intestinal parasitic infections in the community.

In addition to hookworm, *G. lamblia* and *E. histolytica* were found to dominate among the parasites detected in this study. These findings align with reports from studies conducted in Jawi town [42] and Sebeya, Ethiopia [36]. The high prevalence rates of *G. lamblia* and *E. histolytica* infection observed could potentially be attributed to various risk factors. These may include open defecation practices, consumption of raw or unwashed vegetables/fruits, poor hand washing habits before eating, and lack of access to safe drinking water. Transmission of these parasites primarily occurs through the ingestion of contaminated foods or water by healthy individuals. Factors impacting hygiene and water safety could promote increased infection risk.

This study identified the consumption of raw vegetables as a significant predictor of intestinal parasitic infections, demonstrating a positive association between raw vegetable intake and the presence of intestinal parasites. This finding is consistent with studies conducted in Delgi, Ethiopia [50], Bahir Dar City and Mecha District, Ethiopia [12], and Boricha District, South Ethiopia [51], as well as a systematic review in Ethiopia that highlighted foodborne transmission, including raw vegetables, as a major contributor to parasitic infections [26]. The association may be explained by the fact that raw vegetables, especially those irrigated with untreated wastewater or fertilised with fecal matter, are often contaminated with the infective stages of parasites such as *A. lumbricoides* and *G. lamblia* [52, 53]. Contamination may also occur during harvesting, transportation, or marketing if handled in unhygienic conditions [54]. Inadequate washing of vegetables and poor hand hygiene during preparation further increase the risk of ingestion of parasite eggs and cysts [53]. Enhancing food safety practices, such as thoroughly washing vegetables and maintaining proper hand hygiene, could significantly reduce transmission. Furthermore, health education initiatives that raise awareness of the risks associated with consuming raw vegetables and promote safe food preparation behaviours have been shown to decrease the incidence of intestinal parasitic infections [55, 56]. Similarly, children who wore protective shoes irregularly were found to be more frequently infected with intestinal parasites, including hookworm and *S. stercoralis* compared to their peers. Inconsistent shoe use increases vulnerability to soil‐transmitted helminths, as these parasites commonly enter the human body through direct skin contact with contaminated soil This observation aligns with findings from studies conducted in Bahir Dar City and Mecha District, Ethiopia [12], Boricha District, South Ethiopia [51] and a systematic review done in Ethiopia [26]. Similar results have been reported from different parts of Ethiopia [12, 26, 51]. Additionally, studies from Kenya [57], Afghanistan [58], and Thailand [59] confirm that walking barefoot significantly increases the risk of acquiring hookworm and *Strongyloides* infections, particularly among school‐aged children. The increased risk of infection among children who do not wear shoes regularly can be attributed to direct exposure to contaminated soil, where infective larvae are prevalent [60]. Protective footwear serves as a critical barrier against environmental pathogens, and a lack of consistent use can lead to higher rates of infection [58, 60]. Therefore, promoting the regular use of protective shoes among children, coupled with public health education about the risks associated with barefoot exposure, could significantly reduce the incidence of intestinal parasitic infections in vulnerable populations.

Furthermore, students who practiced open defecation faced double the risk of infection compared to those using latrines. This observation is consistent with other research conducted in Sebeya, Ethiopia [36] and a systematic review done in Ethiopia [26]. Similar associations have been observed in studies from other low‐income settings, including Kenya and Bangladesh, where open defecation was linked to significantly higher prevalence of soil‐transmitted helminths [56, 61]. Improper management of human waste can allow disease‐causing pathogens, such as parasite eggs, larvae, and cysts, to contaminate the surrounding environment. Since these transmission stages can spread via the fecal–oral route or through direct skin penetration, exposure to such environmental contamination increases the risk of infection. Implementing proper sanitation facilities and practices could significantly help reduce transmission. Ensuring access to clean latrines, promoting hygienic disposal of human waste, and raising awareness about the health risks associated with open defecation are essential steps in mitigating the incidence of intestinal parasitic infections in the community.

Undernutrition poses a major public health challenge for school‐aged children in developing countries such as Ethiopia, with the potential to impact physical and mental development. The present study found overall undernutrition prevalence among students of 58.3% (95% CI: 53.1–63.0). This rate was higher than previous research conducted on school children in Bahir Dar city (41.6%) [24], Jimma town (21.0%) [62], Mecha district (11.6%) [63], Sekota town (46.3%) [30], Wolaita Zone, South Ethiopia (38.9%) [64], the pooled prevalence of undernutrition in Ethiopia (21.3%) [20], the pooled prevalence of undernutrition in sub‐Saharan African countries (41.34%) [65], and Argentina [65]. This discrepancy may be attributed to differences in the implementation of community health interventions and socioeconomic levels between surveyed family populations. Undernutrition often correlates directly with household monthly income, as lower‐income families may struggle to sufficiently provide children with a balanced diet and meet other nutritional needs. Effective social programs and economic development could help reduce undernutrition rates over time by addressing its social and economic root causes.

The prevalence of wasting, stunting, and underweight was 49.1%, 25.9%, and 14.6%, respectively. The prevalence of wasting in this study was higher than in studies conducted in Gondar town (9.0%) [66], Sekota town [30], and Mecha District, Northwest Ethiopia [63]. Approximately one‐fourth of the school children in the present study were stunted. This finding is lower than studies done in Gondar town, northwest Ethiopia (46.1%) [66] and Arba Minch, Southern Ethiopia (41.9%) [67]. Furthermore, the prevalence of underweight was lower than studies reported in different parts of Ethiopia [12, 24, 30]. These differences might be associated with the source populations studied, types and frequencies of foods, and the economic and social factors within the studied communities.

Meal frequency and infection with intestinal parasites were identified as predictors of undernutrition, consistent with studies in different parts of Ethiopia [12, 24, 30]. Children living in areas where there are problems of food shortages or low family income have a higher chance of being stunted and underweight. The levels of family income dictate the provision of a balanced diet as well as the frequency of meals to their children. Long‐term exposure to food shortages at an early stage of childhood leads to chronic malnutrition, such as stunting. The level of meal frequency during early childhood plays a vital role in physical growth and development [68]. On the other hand, intestinal parasitic infection competes with children for nutritional intake and impairs host immune function, making children more susceptible to diseases [69]. Addressing both malnutrition risks and their underlying socioeconomic drivers could help improve child health outcomes.

### 4.1 Limitations

This study had some limitations. Intestinal parasite intensity quantification and opportunistic intestinal parasites analysis were not performed. Additionally, children's micronutrient intake levels were not determined. Assessing these factors may have offered greater insight into relationships with malnutrition. Future similar research could help address these limitations by measuring infection intensity, evaluating coinfections, and considering micronutrient status. Addressing methodological constraints will strengthen the ability to inform targeted public health interventions.

## 5 Conclusions

The present study revealed that intestinal parasitic infections and undernutrition were serious health problems in school children in the study areas. The habit of eating raw vegetables and unwashed fruits, irregular wearing of closed shoes, and open‐field defecation were the major predisposing factors for intestinal parasitic infections. Meanwhile, meal frequency and intestinal parasitic infection correlated with undernutrition. Therefore, concerted efforts are needed to address the risk factors of intestinal parasitic infection and undernutrition.

## Author Contributions

**Abera Ademasu Birhanu:** conceptualization, data curation, formal analysis, visualization, methodology, project administration, supervision, investigation, software, validation, funding acquisition, resources, writing – original draft. **Mulat Yimer:** conceptualization, data curation, formal analysis, visualization, supervision, writing – original draft, methodology, investigation, project administration, software, validation, funding acquisition, resources. **Habtu Debash:** conceptualization, data curation, formal analysis, visualization, writing – original draft, project administration, writing – review and editing, supervision, investigation, methodology, software, validation, funding acquisition, resources. **Megbaru Alemu Abate:** conceptualization, data curation, formal analysis, visualization, writing – review and editing, project administration, investigation, supervision, methodology, software, validation, funding acquisition, resources, writing – original draft. All authors read and approved the final manuscript.

## Ethics Statement

Ethical approval was secured from the Research and Ethical Review Committee of Bahir Dar University, College of Medicine and Health Sciences.

## Consent

Students were involved in the study after receiving written informed consent from their families/guardians and the school principal.

## Conflicts of Interest

The authors declare no conflicts of interest.

## Transparency Statement

The lead author Abera Ademasu Birhanu affirms that this manuscript is an honest, accurate, and transparent account of the study being reported; that no important aspects of the study have been omitted; and that any discrepancies from the study as planned (and, if relevant, registered) have been explained.

## Data Availability

All authors have read and approved the final version of the manuscript, had full access to all of the data in this study, and take complete responsibility for the integrity of the data and the accuracy of the data analysis. The data that support the findings of this study are available on request from the corresponding author. The data are not publicly available due to privacy or ethical restrictions.

## References

[1] World Health Organization , *Global Distribution and Prevalence of Soil Transmitted Helminth Infections* (World Health Organization Key Fact Sheet, 2020).

[2] J. A. Harp , “Parasitic Infections of the Gastrointestinal Tract,” Current Opinion in Gastroenterology 19, no. 1 (2003): 31–36. 15699890 10.1097/00001574-200301000-00005

[3] N. Fauziah , J. K. Aviani , Y. N. Agrianfanny , and S. N. Fatimah , “Intestinal Parasitic Infection and Nutritional Status in Children Under Five Years Old: A Systematic Review,” Tropical Medicine and Infectious Disease 7, no. 11 (2022): 371. 36422922 10.3390/tropicalmed7110371 PMC9697828

[4] L. W. Wong , K. S. Ong , J. R. Khoo , C. Goh , J. W. Hor , and S. M. Lee , “Human Intestinal Parasitic Infection: A Narrative Review on Global Prevalence and Epidemiological Insights on Preventive, Therapeutic and Diagnostic Strategies for Future Perspectives,” Expert Review of Gastroenterology & Hepatology 14, no. 11 (2020): 1093–1105. 32755242 10.1080/17474124.2020.1806711

[5] K. J.‐J. O. Kadio , D. Cissé , A. L. Abro , et al., “Factors Associated With Intestinal Parasites in the Central Prison of Conakry, Guinea,” PAMJ‐One Health 6, no. 10 (2021), 10.11604/pamj-oh.2021.6.10.31165.

[6] A. Gebreyohanns , M. H. Legese , M. Wolde , G. Leta , and G. Tasew , “Prevalence of Intestinal Parasites Versus Knowledge, Attitude and Practices (KAPs) With Special Emphasis to *Schistosoma mansoni* Among Individuals Who Have River Water Contact in Addiremets Town, Western Tigray, Ethiopia,” PLoS One 13, no. 9 (2018): e0204259. 30252865 10.1371/journal.pone.0204259 PMC6155513

[7] M. A. Assemie , D. Shitu Getahun , Y. Hune , et al., “Prevalence of Intestinal Parasitic Infection and Its Associated Factors Among Primary School Students in Ethiopia: A Systematic Review and Meta‐Analysis,” PLoS Neglected Tropical Diseases 15, no. 4 (2021): e0009379. 33905414 10.1371/journal.pntd.0009379 PMC8104388

[8] K. Papier , G. M. Williams , R. Luceres‐Catubig , et al., “Childhood Malnutrition and Parasitic Helminth Interactions,” Clinical Infectious Diseases 59, no. 2 (2014): 234–243. 24704723 10.1093/cid/ciu211

[9] C. Best , N. Neufingerl , L. van Geel , T. van den Briel , and S. Osendarp , “The Nutritional Status of School‐Aged Children: Why Should We Care?,” Food and Nutrition Bulletin 31, no. 3 (2010): 400–417. 20973461 10.1177/156482651003100303

[10] D. Rugg , H. Marais , M. Carael , P. De Lay , and M. Warner‐Smith , “Are We on Course for Reporting on the Millennium Development Goals in 2015?,” JAIDS Journal of Acquired Immune Deficiency Syndromes 52 (2009): S69–S76. 19901629 10.1097/QAI.0b013e3181baec7c

[11] T. Hailegebreil , “Prevalence and Determinants of Undernutrition Among School Children of Ethiopia: A Systematic Review and Meta‐Analysis,” preprint, Research Square, 2019, 10.21203/rs.2.13434/v1.

[12] B. E. Feleke , “Nutritional Status and Intestinal Parasite in School Age Children: A Comparative Cross‐Sectional Study,” International Journal of Pediatrics 2016, no. 1 (2016): 1–8. 10.1155/2016/1962128 PMC5021489 27656219

[13] L. E. Bain , P. K. Awah , N. Geraldine , et al., “Malnutrition in Sub–Saharan Africa: Burden, Causes and Prospects,” Pan African Medical Journal 15, no. 1 (2013): 1–9. 24255726 10.11604/pamj.2013.15.120.2535 PMC3830470

[14] P. Yap , T. Fürst , I. Müller , S. Kriemler , J. Utzinger , and P. Steinmann , “Determining Soil‐Transmitted Helminth Infection Status and Physical Fitness of School‐Aged Children,” Journal of Visualized Experiments: JoVE 66 (2012): 3966. 10.3791/3966 PMC3486755 22951972

[15] M. L. Zonta , P. Cociancic , E. E. Oyhenart , and G. T. Navone , “Intestinal Parasitosis, Undernutrition and Socio‐Environmental Factors in Schoolchildren From Clorinda Formosa, Argentina,” Revista de Salud Pública 21 (2020): 224–231. 10.15446/rsap.V21n2.73692 33027333

[16] Y. Kesete , H. Tesfahiwet , G. Fessehaye , et al., “Assessment of Prevalence and Risk Factors for Intestinal Parasitosis, Malnutrition, and Anemia Among School Children in Ghindae Area, Eritrea,” Journal of Tropical Medicine 2020, no. 1 (2020): 1–11. 10.1155/2020/4230260 PMC7647778 33178289

[17] C. Yenew , “Prevalence Intestinal Parasitic Infections Among Primary School Children in Ethiopia: A Systematic Review and Meta‐Analysis,” Journal of Research in Health Science 19, no. 1 (2019): e‑16.

[18] S. Eshetie , *Prevalence and Associated Risk Factors of Intestinal Parasitic Infections Among Atsedmariam Primary and Junior School Students, Alefa District, Central Gondar Zone, Ethiopia* (Bahir Dar University, 2022), http://ir.bdu.edu.et/handle/123456789/14480.

[19] T. Hailegebriel , “Prevalence of Intestinal Parasitic Infections and Associated Risk Factors Among Students at Dona Berber Primary School, Bahir Dar, Ethiopia,” BMC Infectious Diseases 17 (2017): 362. 28535750 10.1186/s12879-017-2466-x PMC5442677

[20] M. A. Assemie , A. A. Alamneh , D. B. Ketema , et al., “High Burden of Undernutrition Among Primary School‐Aged Children and Its Determinant Factors in Ethiopia; A Systematic Review and Meta‐Analysis,” Italian Journal of Pediatrics 46 (2020): 118. 32847566 10.1186/s13052-020-00881-w PMC7448995

[21] L. Chelkeba , Z. Mekonnen , D. Emana , W. Jimma , and T. Melaku , “Prevalence of Soil‐Transmitted Helminths Infections Among Preschool and School‐Age Children in Ethiopia: A Systematic Review and Meta‐Analysis,” Global Health Research and Policy 7, no. 1 (2022): 9. 35307028 10.1186/s41256-022-00239-1 PMC8935818

[22] M. Alemu , A. Anley , and K. Tedla , “Magnitude of Intestinal Parasitosis and Associated Factors in Rural School Children, Northwest Ethiopia,” Ethiopian Journal of Health Sciences 29, no. 1 (2019): 923–928, 10.4314/ejhs.v29i1.14. 30700960 PMC6341440

[23] N. Negussu , B. Mengistu , B. Kebede , et al., “Ethiopia Schistosomiasis and Soil‐Transmitted Helminthes Control Programme: Progress and Prospects,” Ethiopian Medical Journal 55, no. Suppl 1 (2017): 75–80. 28878432 PMC5582635

[24] T. Hailegebriel , “Undernutrition, Intestinal Parasitic Infection and Associated Risk Factors Among Selected Primary School Children in Bahir Dar, Ethiopia,” BMC Infectious Diseases 18 (2018): 394. 30103696 10.1186/s12879-018-3306-3 PMC6090691

[25] I. Ephi , Ethiopia Mini Demographic and Health Survey 2019: Key Indicators (EPHI and ICF, 2019).

[26] A. Gezaw , W. Melese , B. Getachew , and T. Belachew , “Double Burden of Malnutrition and Associated Factors Among Adolescent in Ethiopia: A Systematic Review and Meta‐Analysis,” PLoS One 18, no. 4 (2023): e0282240. 37043492 10.1371/journal.pone.0282240 PMC10096189

[27] M. Chessbrough , District Laboratory Practice in Tropical Countries, Microbiology‐2 (Cambridge University Press, 2006), 184–186.

[28] F. S. Pimenta , C. M. Oliveira , W. T. Hattori , and K. R. Teixeira , “Agreement Between Subjective Global Nutritional Assessment and the Nutritional Assessment of the World Health Organization,” Jornal de Pediatria 94, no. 6 (2018): 602–608. 29136495 10.1016/j.jped.2017.09.007

[29] World Health Organization , *Guideline: Preventive Chemotherapy to Control Soil‐Transmitted Helminth Infections in At‐Risk Population Groups* (World Health Organization, 2017). 29578660

[30] H. Debash , M. Alemu , and H. Bisetegn , “The Prevalence of Intestinal Parasites, Undernutrition and Their Associated Risk Factors Among School‐Age Children in Sekota Town, Northeast Ethiopia: A Community‐Based Cross‐Sectional Study,” Health Science Reports 6, no. 3 (2023): e1137. 36860204 10.1002/hsr2.1137 PMC9969049

[31] G. Alemu , Z. Aschalew , and E. Zerihun , “Burden of Intestinal Helminths and Associated Factors Three Years After Initiation of Mass Drug Administration in Arbaminch Zuria District, Southern Ethiopia,” BMC Infectious Diseases 18 (2018): 1–8. 30157789 10.1186/s12879-018-3330-3 PMC6114701

[32] E. Mekonnen and N. Sadik , “Prevalence of Human Intestinal Parasitic Infections and Associated Risk Factors Among School Children in Hakim District, Harari Regional State, Eastern Ethiopia,” Advances in Public Health 2024, no. 1 (2024): 9986078.

[33] D. Damtie , B. Sitotaw , S. Menkir , B. Kerisew , and K. Hussien , “Human Intestinal Parasitic Infections: Prevalence and Associated Risk Factors Among Elementary School Children in Merawi Town, Northwest Ethiopia,” Journal of Parasitology Research 2021, no. 1 (2021): 1–10. 10.1155/2021/8894089 PMC7899771 33628472

[34] H. Bisetegn , H. Debash , H. Ebrahim , Y. Erkihun , M. Tilahun , and D. G. Feleke , “Prevalence and Determinant Factors of Intestinal Parasitic Infections and Undernutrition Among Primary School Children in North‐Central Ethiopia: A School‐Based Cross‐Sectional Study,” Journal of Parasitology Research 2023, no. 1 (2023): 1–10. 10.1155/2023/2256910 PMC10033212 36968675

[35] D. Gebretsadik , M. Tesfaye , A. Adamu , and G. Zewde , “Prevalence of Intestinal Parasitic Infection and Its Associated Factors Among School Children in Two Primary Schools in Harbu Town, North East Ethiopia: Cross‐Sectional Study,” Pediatric Health, Medicine and Therapeutics 11, no. 1 (2020): 179–188. 32607051 10.2147/PHMT.S252061 PMC7297451

[36] A. Dessie , T. G. Gebrehiwot , B. Kiros , S. D. Wami , and D. H. Chercos , “Intestinal Parasitic Infections and Determinant Factors Among School‐Age Children in Ethiopia: A Cross‐Sectional Study,” BMC Research Notes 12 (2019): 777. 31779671 10.1186/s13104-019-4759-1 PMC6883565

[37] N. Chege , “The Prevalence of Intestinal Parasites and Associated Risk Factors in School‐Going Children From Informal Settlements in Nakuru Town, Kenya,” Malawi Medical Journal 32, no. 2 (2020): 80–86. 35140844 10.4314/mmj.v32i2.5 PMC8788588

[38] R. Gupta , B. Rayamajhee , S. P. Sherchan , et al., “Prevalence of Intestinal Parasitosis and Associated Risk Factors Among School Children of Saptari District, Nepal: A Cross‐Sectional Study,” Tropical Medicine and Health 48 (2020): 73. 32848503 10.1186/s41182-020-00261-4 PMC7444033

[39] H. Mahmoudvand , E. Badparva , A. K. Khalaf , M. Niazi , M. Khatami , and M. R. Nazer , “Prevalence and Associated Risk Factors of Intestinal Helminthic Infections in Children From Lorestan Province, Western Iran,” Parasite Epidemiology and Control 9 (2020): e00136. 31993514 10.1016/j.parepi.2020.e00136 PMC6976929

[40] B. E. Feleke and T. H. Jember , “Prevalence of Helminthic Infections and Determinant Factors Among Pregnant Women in Mecha District, Northwest Ethiopia: A Cross Sectional Study,” BMC Infectious Diseases 18 (2018): 373. 30081837 10.1186/s12879-018-3291-6 PMC6080381

[41] D. Tegen and D. Damtie , “Prevalence and Risk Factors Associated With Intestinal Parasitic Infection Among Primary School Children in Dera District, Northwest Ethiopia,” Canadian Journal of Infectious Diseases and Medical Microbiology 2021, no. 1 (2021): 1–15. 10.1155/2021/5517564 PMC8478561 34594431

[42] B. Sitotaw , H. Mekuriaw , and D. Damtie , “Prevalence of Intestinal Parasitic Infections and Associated Risk Factors Among Jawi Primary School Children, Jawi Town, North‐West Ethiopia,” BMC Infectious Diseases 19 (2019): 341. 31023271 10.1186/s12879-019-3971-x PMC6485161

[43] E. Tekalign , A. Sebeta , D. Nureye , T. Duguma , and T. Tesfaye , “Intestinal Parasitic Infections Among Children Aged 7–14 Years in Mizan‐Aman City, Southwest Ethiopia: A Community‐Based Cross‐Sectional Study,” Frontiers in Public Health 12 (2024): 1478293. 39776487 10.3389/fpubh.2024.1478293 PMC11703966

[44] M. N. M. Hamad , A. A. Mokhtar , and M. Eltoum , “Prevalence of Intestinal Parasitic Infections Among School Aged Children in Berber locality, River Nile State, Sudan 2017,” Journal of Microbiology & Experimentation 7, no. 2 (2019): 85–86.

[45] W. Richert , D. Kasprowicz , D. Kołodziej , D. Zarudzka , and K. Korzeniewski , “Intestinal Parasitic Infections Among School Children in Northern Madagascar,” Annals of Agricultural and Environmental Medicine 31, no. 4 (2024): 546–551. 39743713 10.26444/aaem/189514

[46] P. C. Hernández , L. Morales , J. Chaparro‐Olaya , et al., “Intestinal Parasitic Infections and Associated Factors in Children of Three Rural Schools in Colombia. A Cross‐Sectional Study,” PLoS One 14, no. 7 (2019): e0218681. 31291262 10.1371/journal.pone.0218681 PMC6619675

[47] K. Shiferaw , T. Tesfay , G. Kalayu , and G. Kiros , “Human Intestinal Parasites: Prevalence and Associated Risk Factors Among Grade School Children in Maksegnit, Northwest Ethiopia,” Journal of Tropical Medicine 2021, no. 1 (2021): 1–6. 10.1155/2021/6694809 PMC8213467 34221027

[48] A. Aemiro , S. Menkir , and A. Girma , “Prevalence of Soil‐Transmitted Helminth Infections and Associated Risk Factors Among School Children in Dembecha Town, Ethiopia,” Environmental Health Insights 18 (2024): 11786302241245851. 38628466 10.1177/11786302241245851 PMC11020722

[49] S. Brooker , J. Bethony , and P. J. Hotez , “Human Hookworm Infection in the 21st Century,” Advances in Parasitology 58 (2004): 197. 15603764 10.1016/S0065-308X(04)58004-1 PMC2268732

[50] A. Ayalew , T. Debebe , and A. Worku , “Prevalence and Risk Factors of Intestinal Parasites Among Delgi School Children, North Gondar, Ethiopia,” Journal of Parasitology and Vector Biology 3, no. 5 (2011): 75–81.

[51] B. Tsegaye , A. Yoseph , and H. Beyene , “Prevalence and Factors Associated With Intestinal Parasites Among Children of Age 6 to 59 Months in, Boricha District, South Ethiopia, in 2018,” BMC Pediatrics 20 (2020): 28. 31969128 10.1186/s12887-020-1935-3 PMC6975076

[52] M. A. Eraky , S. M. Rashed , M. E. S. Nasr , A. M. S. El‐Hamshary , and A. Salah El‐Ghannam , “Parasitic Contamination of Commonly Consumed Fresh Leafy Vegetables in Benha, Egypt,” Journal of Parasitology Research 2014, no. 1 (2014): 1–7. 10.1155/2014/613960 PMC4084512 25024845

[53] T. Asfaw , D. Genetu , D. Shenkute , T. T. Shenkutie , Y. E. Amare , and B. Yitayew , “Parasitic Contamination and Microbiological Quality of Commonly Consumed Fresh Vegetables Marketed in Debre Berhan Town, Ethiopia,” Environmental Health Insights 17 (2023): 11786302231154755. 36798697 10.1177/11786302231154755 PMC9926396

[54] T. Tefera and G. Mebrie , “Prevalence and Predictors of Intestinal Parasites Among Food Handlers in Yebu Town, Southwest Ethiopia,” PLoS One 9, no. 10 (2014): e110621. 25329050 10.1371/journal.pone.0110621 PMC4201565

[55] World Health Organization, “Guidelines on Sanitation and Health,” In *Guidelines on Sanitation and Health* (World Health Organization, 2018), 220–220.

[56] E. C. Strunz , D. G. Addiss , M. E. Stocks , S. Ogden , J. Utzinger , and M. C. Freeman , “Water, Sanitation, Hygiene, and Soil‐Transmitted Helminth Infection: A Systematic Review and Meta‐Analysis,” PLoS Medicine 11, no. 3 (2014): e1001620. 24667810 10.1371/journal.pmed.1001620 PMC3965411

[57] M. Freeman , A. N. Chard , B. Nikolay , et al., “Associations Between School‐ and Household‐Level Water, Sanitation and Hygiene Conditions and Soil‐Transmitted Helminth Infection Among Kenyan School Children,” Parasites & Vectors 8 (2015): 1–13. 26248869 10.1186/s13071-015-1024-x PMC4528701

[58] B. A. Rahimi , B. A. Mahboobi , M. H. Wafa , M. S. Sahrai , M. H. Stanikzai , and W. R. Taylor , “Prevalence and Associated Risk Factors of Soil‐Transmitted Helminth Infections in Kandahar, Afghanistan,” BMC Infectious Diseases 22, no. 1 (2022): 361. 35410154 10.1186/s12879-022-07336-z PMC9003950

[59] V. Jiraanankul , W. Aphijirawat , M. Mungthin , et al., “Incidence and Risk Factors of Hookworm Infection in a Rural Community of Central Thailand,” American Society of Tropical Medicine and Hygiene 84, no. 4 (2011): 594–598. 10.4269/ajtmh.2011.10-0189 PMC3062455 21460016

[60] P. J. Hotez and A. Kamath , “Neglected Tropical Diseases in Sub‐Saharan Africa: Review of Their Prevalence, Distribution, and Disease Burden,” PLoS Neglected Tropical Diseases 3, no. 8 (2009): e412. 19707588 10.1371/journal.pntd.0000412 PMC2727001

[61] K. Ziegelbauer , B. Speich , D. Mäusezahl , R. Bos , J. Keiser , and J. Utzinger , “Effect of Sanitation on Soil‐Transmitted Helminth Infection: Systematic Review and Meta‐Analysis,” PLoS Medicine 9, no. 1 (2012): e1001162. 22291577 10.1371/journal.pmed.1001162 PMC3265535

[62] Z. Mekonnen , D. Hassen , S. Debalke , et al., “Soil‐Transmitted Helminth Infections and Nutritional Status of School Children in Government Elementary Schools in Jimma Town, Southwestern Ethiopia,” SAGE Open Medicine 8 (2020): 2050312120954696. 32953118 10.1177/2050312120954696 PMC7475784

[63] T. Tewabe and A. Belachew , “Determinants of Nutritional Status in School‐Aged Children in Mecha, Northwest Ethiopia,” Current Therapeutic Research 93 (2020): 100598. 32939225 10.1016/j.curtheres.2020.100598 PMC7476852

[64] D. K. Meskele , T. L. Abiso , T. B. Belete , M. M. Koyira , and S. K. Dake , “Undernutrition and Associated Factors Among School‐Age Children in Wolaita Zone, South Ethiopia: A Comparative Cross‐Sectional Study,” Frontiers in Nutrition 11 (2024): 1400276. 39360282 10.3389/fnut.2024.1400276 PMC11445009

[65] B. Mengistu , A. K. Belew , L. D. Baffa , et al., “Prevalence of Undernutrition Among Children and Adolescents With Cancer Living in Sub‐Saharan African Countries: A Systematic Review and Meta‐Analysis,” Global Pediatric Health 11 (2024): 2333794X241298807. 10.1177/2333794X241298807 PMC11590137 39600491

[66] Z. Getaneh , M. Melku , M. Geta , T. Melak , and M. T. Hunegnaw , “Prevalence and Determinants of Stunting and Wasting Among Public Primary School Children in Gondar Town, Northwest, Ethiopia,” BMC Pediatrics 19 (2019): 207. 31238889 10.1186/s12887-019-1572-x PMC6591879

[67] E. Z. Tariku , G. A. Abebe , Z. A. Melketsedik , and B. T. Gutema , “Prevalence and Factors Associated With Stunting and Thinness Among School‐Age Children in Arba Minch Health and Demographic Surveillance Site, Southern Ethiopia,” PLoS One 13, no. 11 (2018): e0206659. 30388149 10.1371/journal.pone.0206659 PMC6214544

[68] S. Moradi , A. Mirzababaei , H. Mohammadi , et al., “Food Insecurity and the Risk of Undernutrition Complications Among Children and Adolescents: A Systematic Review and Meta‐Analysis,” Nutrition 62 (2019): 52–60. 30852458 10.1016/j.nut.2018.11.029

[69] L. S. Stephenson , M. C. Latham , and E. A. Ottesen , “Malnutrition and Parasitic Helminth Infections,” Parasitology 121, no. S1 (2000): S23–S38. 11386688 10.1017/s0031182000006491

